# Endovascular Management of Extra-Peritoneal Hematoma Secondary to Bladder Catheterization

**DOI:** 10.7759/cureus.39256

**Published:** 2023-05-20

**Authors:** Arnaud Bourguignon, Lea Tannouri, Ilias Bennouna, Ryan El Amine, Salvatore Murgo

**Affiliations:** 1 Radiology, Hôpital Universitaire de Bruxelles, Brussels, BEL; 2 Interventional Radiology, Hospital Erasme, Brussels, BEL

**Keywords:** urological trauma, foley catheterization, interventional radiology guided embolization, bladder rupture, hemorrhage

## Abstract

Bladder rupture related to Foley catheter insertion is a rare condition mainly reported in patients with chronic bladder disease. In the present case, this rare condition was associated with massive hematoma due to active arterial bleeding, which was treated by embolization. We present the case of a 38-year-old woman admitted to the gastroenterology department with decompensated alcoholic liver cirrhosis, anemia, malnutrition, and diabetes. Six days after admission, she presented with hypotension and tachycardia associated with gross hematuria. An abdominal computed tomography scan revealed a Foley catheterization-related bladder perforation and a massive extra-peritoneal hematoma caused by active arterial bleeding from a distal branch of the right vesical artery. A successful embolization was performed with microparticles and coiled with complete hemorrhage control on post-procedure imaging. The bladder perforation was treated conservatively with a urinary drainage catheter, irrigation, and antibiotics. Despite these measures, the patient died 15 days later due to liver failure and sepsis. Our case highlights that commonly performed simple procedures can lead to severe complications, especially in frail patients.

## Introduction

Although rare, bladder rupture is usually due to either abdominal or pelvic blunt trauma but also may be iatrogenic, related to surgical or endoscopic procedures [1]. Very rare cases are related to Foley catheter insertion, mainly in patients with chronic bladder disease [2]. Morbidity and mortality associated with bladder rupture are high, and treatment is either surgical or conservative, with drainage and lavage [3]. We report here a case of iatrogenic perforation with active bleeding for which radiological embolization played a major role in attempting conservative management.

## Case presentation

**Figure 1:**
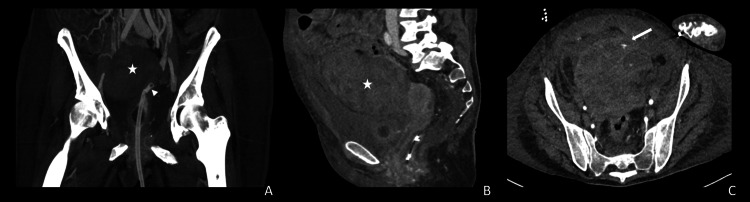
Abdominal CT scan. A) Maximal intensity projection on a coronal plan. B) Portal phase - sagittal plan . C) Arterial phase - axial plan. These demonstrate the bladder perforation due to the foley catheter (white arrowhead) leading to active arterial bleeding (white arrow) and a significant extra-peritoneal hematoma (white star).

**Figure 2:**
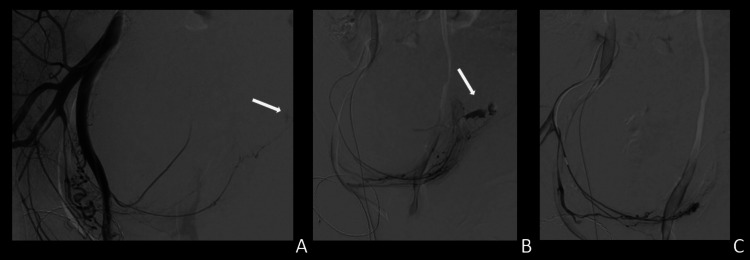
Angiography and embolization. A and B) Active hemorrhage (white arrow) from the distal part of the vesical artery. C) Post-embolization control.

A 38-year-old woman was admitted into the gastro-enterology department with decompensated alcoholic liver cirrhosis (encephalopathy, hyperbilirubinemia [21 mg/dL], coagulopathy), anemia, malnutrition, and diabetes. Initial treatment included nutritional support, vitamin supplementation, and limited blood transfusion. Transjugular liver biopsy confirmed alcoholic hepatitis (Maddrey score: 54). At day three post-admission, a bladder globe was diagnosed, and a urinary catheter was placed. Three days after, the patient presented with hypotension and tachycardia associated with nausea, vomiting, abdominal distension, confusion, and macroscopic hematuria. Hemoglobin levels dropped to 4.8 g/dL, and platelets dropped to 66,000/mm^3^. An abdominal computed tomography (CT) scan revealed a bladder perforation and a massive extra-peritoneal hematoma secondary to active arterial bleeding (Figure 1). The patient was referred to the interventional radiology department after a multidisciplinary decision, the urgent surgical option being discarded due to the patient's clinical status and severe comorbidities. After ultrasound-guided puncture of the right common femoral artery, the placement of a 4 French (Fr) sheath according to the Seldinger technique and selective catheterization of the right hypogastric artery with a C2 Cobra (Cordis, Florida, USA) catheter, arteriography was performed and allowed the identification of the precise origin of the active arterial bleeding from a distal branch of the right vesical artery (Figure 2A). This branch was selectively catheterized, and an additional contrast injection was performed further to confirm the bleeding site (Figure 2B). A successful embolization was then performed using a 2.7Fr Progreat microcatheter (Terumo, Tokyo, Japan) with microparticles (300-500 Microns) and coils resulting in complete control of the hemorrhage on post-procedure imaging (Figure 2C). The bladder perforation was conservatively managed with a urinary drainage catheter, irrigation, and antibiotics. Despite these life-saving measures, the patient died 15 days later due to liver failure and ongoing sepsis.

## Discussion

Although urinary catheter placement is considered a simple and safe procedure commonly performed in healthcare settings, our case illustrates the worst complication, bladder perforation with arterial bleeding due to a lesion of the vesical artery [4]. Angiographic embolization, performed after selective catheterization of the bleeding artery in order to avoid non-target embolization and possible ischemia, provided control of the bleeding. However, sepsis associated with liver failure was the cause of death in this very fragile patient. This case may serve as a reminder that urinary catheter placement is not such a benign procedure, especially in patients with advanced liver disease. Other complications of ureteral catheterization include iatrogenic urethral lesions, bleeding, and urinary tract infection [5]. Risk factors of such complications include age, the training level of the practitioner, patient comorbidities, the presence of prostate hyperplasia, the duration of the catheterization, and the presence of bladder wall lesions. Indeed, irradiation, bladder tumors, chronic inflammation, and/or infection can lead to weakened bladder walls [2,3]. Aside from severe liver disease, none of the above-mentioned risk factors were present in our case, and perforation was probably related, at least in part, to technically inadequate insertion. Bladder rupture is a rare condition representing 1.6% of patients with abdominal trauma (60% extra-peritoneal, 30% intraperitoneal, and 10% both) [6,7].

Multiple causes have been highlighted, including: 1) Spontaneous ruptures, which are rare but associated with high mortality. These may be related to vaginal delivery, hemophilia, neoplasia, irradiation, infection, alcohol intoxication (increased urine volume and decreased perception of desire to void), and urinary retention. 2) Traumatic ruptures are often due to pelvic fractures after falls, motor vehicle crashes, or penetrating wounds. 3) Iatrogenic ruptures after surgery, urologic procedures, bladder punctures, or, as in our case, after a Foley catheter placement [8]. Clinical presentations of bladder rupture include hematuria, abdominal tenderness, renal insufficiency, ascites, sepsis, urinary disorders, and inability to empty the bladder [6] but vary according to the bladder perforation type [9]. In our case, the rupture was extra-peritoneal and the hematoma, making it a type 4 perforation [10]. According to the literature, surgical management is recommended in cases of intraperitoneal bladder perforation, while a conservative approach is favored for other (retroperitoneal or mixed) bladder perforation types [2,8-13]. Active bleeding associated with bladder perforation is an even rare condition, and conservative management was justified by the extra-peritoneal localization of the perforation and the hematoma, the precise control of the arterial bleeding, and the patient's comorbidities. These comorbidities and the presence of difficult-to-manage sepsis were the major cause of death, although local control of bladder perforation had been achieved. Finally, selective percutaneous, trans-catheter, arterial embolization (TAE) of bladder arteries is a life-saving procedure that has very satisfactory technical success, periprocedural morbidity and mortality rates, and sustained long-term control of haematuria with low recurrence rates [14]. It is also less invasive than surgical procedures and should therefore be considered an alternative treatment option in selected patients.

## Conclusions

Our case illustrates an example of the worst complication after simple urinary catheter placement: bladder perforation with arterial bleeding. Selective TAE of bladder arteries is a safe technique that provides bleeding control, while sepsis associated with liver failure was the cause of death in this very fragile patient. This case should serve as a reminder that even a simple urinary catheter placement can lead to severe complications, especially in high-risk patients (such as our patient with advanced liver disease).

## References

[1] Poola S, Mohan A (2018). A Foley fallacy: a case of bladder rupture after “routine” Foley catheter placement. Case Rep Urol.

[2] Ogawa S, Date T, Muraki O (2013). Intraperitoneal urinary bladder perforation observed in a patient with an indwelling urethral catheter. Case Rep Urol.

[3] Alzughaibi M, Althonayan N, Alrabeeah K, Alkhayal A (2022). Foley catheter erosion through bladder wall causing intraperitoneal bladder injury: a case report. J Surg Case Rep.

[4] Raheem OA, Jeong YB (2011). Intraperitoneally placed Foley catheter via verumontanum initially presenting as a bladder rupture. J Korean Med Sci.

[5] Kim IY, Lee SB, Choi BK (2012). Bladder rupture in immediate postrenal transplant period of uncertain cause. Exp Clin Transplant.

[6] Simon LV, Sajjad H, Lopez RA, Burns B (2023). Bladder Rupture. http://www.ncbi.nlm.nih.gov/books/NBK470226/.

[7] Morey AF, Brandes S, Dugi DD 3rd (2014). Urotrauma: AUA guideline. J Urol.

[8] Zhang Y, Yuan S, Alshayyah RW (2021). Spontaneous rupture of urinary bladder: two case reports and review of literature. Front Surg.

[9] Vaccaro JP, Brody JM (2000). CT cystography in the evaluation of major bladder trauma. Radiographics.

[10] Tabaru A, Endou M, Miura Y, Otsuki M (1996). Generalized peritonitis caused by spontaneous intraperitoneal rupture of the urinary bladder. Intern Med.

[11] Geng JH, Chang HC, Chung SD (2014). Nonoperative treatment for intraperitoneal bladder rupture. Urol Sci.

[12] Bryk DJ, Zhao LC (2016). Guideline of guidelines: a review of urological trauma guidelines. BJU Int.

[13] Morey AF, Broghammer JA, Hollowell CM, McKibben MJ, Souter L (2021). Urotrauma guideline 2020: AUA guideline. J Urol.

[14] Tsitskari M, Spiliopoulos S, Konstantos C, Palialexis K, Reppas L, Brountzos E (2020). Long-term results of super-selective trans-catheter embolization of the vesical arteries for the treatment of intractable bladder haematuria. CVIR Endovasc.

